# Joint Parameter Estimation for the Two-Wave with Diffuse Power Fading Model

**DOI:** 10.3390/s16071014

**Published:** 2016-06-30

**Authors:** Jesus Lopez-Fernandez, Laureano Moreno-Pozas, Francisco Javier Lopez-Martinez, Eduardo Martos-Naya

**Affiliations:** Departamento de Ingeniería de Comunicaciones, ETS Ingeniería de Telecomunicación, Universidad de Málaga, Málaga 29071, Spain; lmp@ic.uma.es (L.M.-P.); fjlopezm@ic.uma.es (F.J.L.-M.); eduardo@ic.uma.es (E.M.-N.)

**Keywords:** Cramer-Rao bound, moment-based estimation, fading channels, Rician fading, Two-Wave with diffuse power

## Abstract

Wireless sensor networks deployed within metallic cavities are known to suffer from a very severe fading, even in strong line-of-sight propagation conditions. This behavior is well-captured by the Two-Wave with Diffuse Power (TWDP) fading distribution, which shows great fit to field measurements in such scenarios. In this paper, we address the joint estimation of the parameters *K* and Δ that characterize the TWDP fading model, based on the observation of the received signal envelope. We use a moment-based approach to derive closed-form expressions for the estimators of *K* and Δ, as well as closed-form expressions for their asymptotic variance. Results show that the estimation error is close to the Cramer-Rao lower bound for a wide range of values of the parameters *K* and Δ. The performance degradation due to a finite number of observations is also analyzed.

## 1. Introduction

Rician fading model is extensively used to characterize the random fluctuations of the received signal amplitude in line-of-sight (LOS) environments [1]. In such scenarios, the scattering waves arriving at the receiver can be split into the dominant LOS component plus a diffuse component, which accounts for the effect of the non-LOS (NLOS) propagation. The relative strength of the LOS component with respect to the NLOS one is measured by the Rician *K*-factor, defined as the ratio between the powers of both components.

The estimation of the Rician *K*-factor is of paramount importance in the context of wireless communications systems, as the proper system operation heavily relies on the quality of the estimation of *K* [2,3]. As a matter of fact, a flurry of papers have addressed this estimation problem from different perspectives [4,5,6,7,8] in the last years.

However, in some indoor and outdoor LOS environments the Rician distribution falls short to accurately modeling small-scale fading effects. This is of special relevance in the case of wireless sensor networks (WSN) deployed on the inner surface of cavities (e.g., tunnels, plane and helicopter airframes, buses, shipping containers) used to measure data for maintenance, comfort, health and security purposes [9,10,11] or in vehicle-to-vehicle links [12], or in general, machine-to-machine systems like WSN deployed inside and/or around complex structures. In all the aforementioned scenarios, field measurements show that fading in the presence of LOS components may even degenerate into a regime more severe than Rayleigh fading [9,10,11,12].

Among the different distributions in the literature which may be useful to account for this special propagation regime, the Two-Wave with diffuse power (TWDP) fading model is the preferred alternative because of its flexibility [11] and clear physical interpretation. This fading model was first presented in [13] as a generalization of the Rician fading model, by incorporating a second dominant LOS component with uniformly distributed phase. The effect of this new LOS component is captured through the parameter Δ, which measures the relative magnitudes of the two LOS components to one another. This model has succeeded on characterizing a wide variety of fading behaviors, from purely Rician to worse than Rayleigh fading [14,15], and has been considered by many authors [16,17,18,19] in order to evaluate the performance of communication systems operating under this peculiar fading condition.

Despite its relevance, the estimation of the parameters *K* and Δ for the TWDP fading model has not been addressed in the literature to the best of our knowledge, except for the preliminary results in [20] based on maximum-likelihood estimation. Thence, we aim to fill this gap by studying the problem of jointly estimating *K* and Δ from the observation of the received signal envelope. Specifically, we design a moment-based estimator for the TWDP fading parameters leveraging the closed-form expressions of the moments recently proposed in [15]. We also derive the asymptotic variance of the estimator, and compare it to the Cramer-Rao lower bound (CRB) via Monte Carlo simulations. The effect of a finite number of observations on the estimator performance is also quantified.

The remainder of this paper is structured as follows: Section 2 introduces the main aspects of the TWDP fading model, which will be of use in the following derivations. The parameter estimation is addressed in Section 3, whereas the CRB and the asymptotic variance of the proposed estimators are presented in Section 4. The effect of having a finite number of observations in the estimation performance is studied in Section 5, and the main conclusions are outlined in Section 6.

## 2. TWDP Fading Model

In the TWDP fading model, the complex baseband signal $V_{r}$ at the receiver side can be expressed as ([13] Equation (4))

(1) $$V_{r}=V_{1}\exp\left(j\phi_{1}\right)+V_{2}\exp\left(j\phi_{2}\right)+V_{d}$$

We observe two LOS components with uniformly distributed phases $\phi_{1},\;\phi_{2}∼U\left(0,2\pi \right)$ (The symbol ∼ reads as *statistically distributed as*.) and constant amplitudes $V_{1}$ and $V_{2}$, plus a diffuse component $V_{d}=X+jY$ regarded as a complex Gaussian random variable, being $X,\;Y∼N(0,\sigma^{2})$ and independent. The TWDP model is fully characterized by the parameters *K*, Δ and Ω defined as

(2) $$K=\frac{V_{1}^{2}+V_{2}^{2}}{2\sigma^{2}},\;\Delta =\frac{2V_{1}V_{2}}{V_{1}^{2}+V_{2}^{2}},\;\Omega =V_{1}^{2}+V_{2}^{2}+2\sigma^{2}$$

The parameter K∈[0,∞) has an equivalent interpretation as in the Rician case, whereas the parameter Δ∈[0,1] indicates whether the two LOS components have equal amplitude (Δ=1) or not (Δ<1), degenerating in the Rician fading model for Δ=0. The pdf of $r=|V_{r}|$ is given by ([13] Equation (32))

(3) $$\begin{array}{cc} f(r) & =\frac{r}{2\pi \sigma^{2}}e^{-\frac{r^{2}}{2\sigma^{2}}-K}\int_{0}^{2\pi }e^{K\Delta \cos(\theta )}I_{0}\left(\frac{r}{\sigma }c_{\theta }\right)d\theta \end{array}$$

where $I_{0}\left(\cdot \right)$ is the modified Bessel function of the first kind and order zero, and $c_{\theta }=\sqrt{2K[1+\Delta \cos(\theta )]}$. Finally, the parameter Ω corresponds to the average received signal power and hence is merely a scale factor for the model. Although we focus our study exclusively in the estimation of the K and Δ parameters, the parameter Ω is also unknown and related to *K* and Δ and must therefore be estimated as well.

In ([15] Equation (30)), the moments of the squared signal envelope $\gamma =r^{2}$ were calculated as a finite-range integral involving the Kummer confluent hypergeometric function ${}_{1}F_{1}\left(\cdot ,\cdot ;\cdot \right)$. Using the connection between the Kummer function and the Laguerre polynomials $L_{k}\left(\cdot \right)$ [21] [Equation 8.972], we have

(4) $$E\left(\gamma^{k}\right)=\frac{k!\Omega^{k}}{{\left(1+K\right)}^{k}2\pi }\int_{0}^{2\pi }L_{k}\left(-K(1+\Delta \cos\theta )\right)d\theta$$

Noting that the Laguerre polynomials can be expressed as [21] [Equation 8.970]

(5) Lk(-x)=∑m=0kkmxmm!

the moments can be computed in closed-form by direct integration of Equation (4) as

(6) E(γk)=k!Ωk(1+K)k2π∑m=0kkmKmm!∫02π(1+Δcosθ)mdθ

by using a simple change of variables t=cosθ. Besides, the first moments of γ coincide with the first even moments of $r^{2}$, and can be computed from Equation (6) as

(7) $$\begin{array}{cc} E(\gamma ) & =\mu_{2}=\Omega \end{array}$$

(8) $$\begin{array}{cc} E(\gamma^{2}) & =\mu_{4}=\frac{\Omega^{2}\left(4+K\left(8+K\left(2+\Delta^{2}\right)\right)\right)}{2{\left(1+K\right)}^{2}} \end{array}$$

(9) $$\begin{array}{cc} E(\gamma^{3}) & =\mu_{6}=\frac{\Omega^{3}\left(12+K\left(3+K\right)\left(12+K\left(2+3\Delta^{2}\right)\right)\right)}{2{\left(1+K\right)}^{3}} \end{array}$$

where $\mu_{n}$ is the *n*-th order moment of the received signal envelope, i.e., $\mu_{n}=E\left[r^{n}\right]$. The second, fourth and sixth-order moments $\mu_{2}$, $\mu_{4}$, $\mu_{6}$ are expressed in closed-form in terms of the parameters *K* and Δ and Ω. We will use these expressions to design a moment-based joint estimator.

## 3. Moment-Based Estimation of *K* and Δ

Dividing Equation (8) by $\mu_{2}^{2}$ and Equation (9) by $\mu_{2}^{3}$, we obtain

(10) $$\begin{array}{cc} \frac{\mu_{4}}{\mu_{2}^{2}} & =\frac{4+K(8+K\left(2+\Delta^{2}\right))}{2{\left(1+K\right)}^{2}} \end{array}$$

(11) $$\begin{array}{cc} \frac{\mu_{6}}{\mu_{2}^{3}} & =\frac{12+K\left(3+K\right)(12+K\left(2+3\Delta^{2}\right))}{2{\left(1+K\right)}^{3}} \end{array}$$

Using the sample moments ${\hat{\mu }}_{n}=\frac{1}{N}\sum_{l=1}^{N}r_{l}^{n}$ instead of the ensemble averages $\mu_{n}$, Equations (10) and (11) can be solved for *K* and Δ resulting in moment-based estimators (A similar procedure is used in [6] for designing moment-based estimators of the Rician K factor where a function to denote the ratio of different moments is introduced and denoted as $f_{n,m}$. The terms $\frac{\mu_{4}}{\mu_{2}^{2}}$ and $\frac{\mu_{6}}{\mu_{2}^{3}}$ on the left hand side of Equations (10) and (11) would correspond to $\sqrt{f_{4,2}}$ and $\sqrt{f_{6,2}}$ respectively.). In this procedure notice that the third parameter Ω is also being estimated when using ${\hat{\mu }}_{2}$ instead of $\mu_{2}$ as indicated in Equation (7). Solving Equation (10) for Δ and substituting it in Equation (11) results in the cubic polynomial equation on $\hat{K}$

(12) $${\hat{K}}^{3}+a_{1}{\hat{K}}^{2}+a_{2}\hat{K}+a_{3}=0$$

with coefficients

(13) $$\begin{array}{cc} a_{1} & =\frac{6{\hat{\mu }}_{6}-30{\hat{\mu }}_{4}{\hat{\mu }}_{2}+24{\hat{\mu }}_{2}^{3}}{2{\hat{\mu }}_{6}-6{\hat{\mu }}_{4}{\hat{\mu }}_{2}+4{\hat{\mu }}_{2}^{3}}\\ a_{2} & =\frac{6{\hat{\mu }}_{6}-42{\hat{\mu }}_{4}{\hat{\mu }}_{2}+48{\hat{\mu }}_{2}^{3}}{2{\hat{\mu }}_{6}-6{\hat{\mu }}_{4}{\hat{\mu }}_{2}+4{\hat{\mu }}_{2}^{3}}\\ a_{3} & =\frac{2{\hat{\mu }}_{6}-18{\hat{\mu }}_{4}{\hat{\mu }}_{2}+24{\hat{\mu }}_{2}^{3}}{2{\hat{\mu }}_{6}-6{\hat{\mu }}_{4}{\hat{\mu }}_{2}+4{\hat{\mu }}_{2}^{3}} \end{array}$$

The closed-form solution of a cubic polynomial results in the estimator

(14) $$\hat{K}={\left(p+{\left(p^{2}+q^{3}\right)}^{\frac{1}{2}}\right)}^{\frac{1}{3}}+{\left(p-{\left(p^{2}+q^{3}\right)}^{\frac{1}{2}}\right)}^{\frac{1}{3}}-\frac{a_{1}}{3}$$

with

(15) $$\begin{array}{cc} p & =\frac{1}{54}\left(9a_{1}a_{2}-27a_{3}-2a_{1}^{3}\right),\\ q & =\frac{1}{9}\left(3a_{2}-a_{1}^{2}\right) \end{array}$$

Note that, while Equation (12) has three possible solutions, only one of them yields a valid estimation of *K* (i.e., real and positive); this is the solution stated in Equation (14). Plugging the estimate of *K* in Equation (10) yields a quadratic polynomial in Δ, for which the following estimator is obtained:

(16) $$\hat{\Delta }=\frac{\sqrt{2\frac{{\hat{\mu }}_{4}}{{\hat{\mu }}_{2}^{2}}{\left(1+\hat{K}\right)}^{2}-2{\hat{K}}^{2}-8\hat{K}-4}}{\hat{K}}$$

where the positive-valued solution has been selected, as Δ≥0 by definition.

## 4. Asymptotic Variance and Cramer-Rao Bound

To assess the performance of the proposed estimators, we will first calculate the asymptotic variance. Then, we will compare it to the CRB bound, which determines the minimum achievable variance of any unbiased estimator.

It is well-known that for any moment-based estimator, as the number of independent and identically distributed (i.i.d.) observations *N* increases, the estimator bias tends asymptotically to zero. Similarly, in this situation the estimator variance tends to vary proportionally to 1/N (it is a $\sqrt{N}$-consistent estimator). In our problem, the proposed estimators are a function of three sample moments, i.e., $\hat{K}=h_{1}\left({\hat{\mu }}_{2},{\hat{\mu }}_{4},{\hat{\mu }}_{6}\right)$ and $\hat{\Delta }=h_{2}\left({\hat{\mu }}_{2},{\hat{\mu }}_{4},{\hat{\mu }}_{6}\right)$ where the function $h_{1}\left(\cdot \right)$ is obtained by substituting Equations (13) and (15) in Equation (14) and the function $h_{2}\left(\cdot \right)$ is obtained by substituting Equation (14) in Equation (16). The asymptotic variance for the estimation of *K* is given by [22] [Equation 9.16]:

(17) $${\text{AsV}}_{K}=g_{K}\Sigma \;g_{K}^{T}$$

where $g_{K}$ is the vector of derivatives of *K* with respect to each sample moment, evaluated at the corresponding statistical moment value, i.e.,

(18) $$g_{K}={\left.\left[\frac{\partial \hat{K}}{\partial {\hat{\mu }}_{2}},\frac{\partial \hat{K}}{\partial {\hat{\mu }}_{4}},\frac{\partial \hat{K}}{\partial {\hat{\mu }}_{6}}\right]\right|}_{{\hat{\mu }}_{2}=\mu_{2},{\hat{\mu }}_{4}=\mu_{4},{\hat{\mu }}_{6}=\mu_{6}}$$

and **Σ** is the 3 × 3 covariance matrix of the ensemble averages whose entries are defined as ${\left[\Sigma \right]}_{ij}=\text{cov}\left({\hat{\mu }}_{2i},{\hat{\mu }}_{2j}\right),i,j=1,2,3$. It can be easily shown that

(19) $${\left[\Sigma \right]}_{ij}=\frac{1}{N}\left(\mu_{2i+2j}-\mu_{2i}\mu_{2j}\right),\;\;\;\;\;\;\;\;\;\;i,j=1,2,3$$

Evaluation of Equation (19) requires the use of all the even order moments up to order 12. The first three moments are shown in Equations (7)–(9). Closed-form expressions for $\mu_{8},\mu_{10}$ and $\mu_{12}$ can be easily obtained by evaluating Equation (6), and are shown in Appendix A. Replacing *K* by Δ in Equations (17) and (18) yields the corresponding expression for $AsV_{\Delta }$.

The Cramer-Rao lower bound is directly related to the Fisher Information Matrix (FIM), which will be denoted by I(θ) with entries ${\left[I\left(\theta \right)\right]}_{ij}$ where θ=[K,Δ,Ω] is the parameter vector to be estimated. For i.i.d. observations the FIM matrix is a 3 × 3 matrix with

(20) $${\left[I\left(\theta \right)\right]}_{ij}=N\;\;E{\left[\frac{\partial \ln f(r)}{\partial \theta_{i}}\cdot \frac{\partial \ln f(r)}{\partial \theta_{j}}\right]}_{i,j=1,2,3}$$

where *N* is the number of observations and f(r) is the TWDP pdf in Equation (3).

The CRBs for the estimation of *K* and Δ will be denoted here as ${\text{CRB}}_{K}$ and ${\text{CRB}}_{\Delta }$ respectively. According to [22] , these can be expressed as the elements (1,1) and (2,2) of the inverse FIM matrix, respectively, i.e., ${\text{CRB}}_{K}={\left[I{\left(\theta \right)}^{-1}\right]}_{11}$ and ${\text{CRB}}_{\Delta }={\left[I{\left(\theta \right)}^{-1}\right]}_{22}$. See that the CRBs decrease with the number of observations as 1/N. In order to compute the FIM entries we must first obtain the derivatives in Equation (20), and then perform numerical integration. As stated before, although we are interested in the estimation of *K* and Δ, the parameter Ω must likewise be estimated and accounted for in the CRB calculation. Whether Ω is known or not indeed affects the CRB, but the specific value of Ω is irrelevant because the CRB is scale invariant.

Before presenting the results for CRB and AsV, we would like to remark an important aspect relative to normalization. In the context of moment-based estimators it is common (see Figure 2 in [6]) to normalize the AsV and the CRB by *N* because the (asymptotic) behavior of the proposed estimator when N is high can be seen in one single figure and compared to the CRB. Therefore in the sequel, results for both the CRB and the AsV will be normalized with respect to *N*. A further normalization with respect to the estimated parameter true value will be carried out in order to have bounds on *relative* errors (instead of *absolute* errors) which give better information of the goodness of the estimation. Finally, it is usual in the literature to represent the bound on the standard deviation of the error instead of on the variance. Hence, the square root of CRB and AsV will be considered. For the sake of compactness, in the text we will refer to the square root of the CRB for the estimation of K, normalized to both *K* and *N* as sqrt-normalized ${\text{CRB}}_{K}$ which stands for $\sqrt{\frac{{\text{CRB}}_{K}N}{K^{2}}}$ and the same holds for the sqrt-normalized ${\text{CRB}}_{\Delta }$, ${\text{AsV}}_{K}$ and ${\text{AsV}}_{\Delta }$.

**Figure 1:**
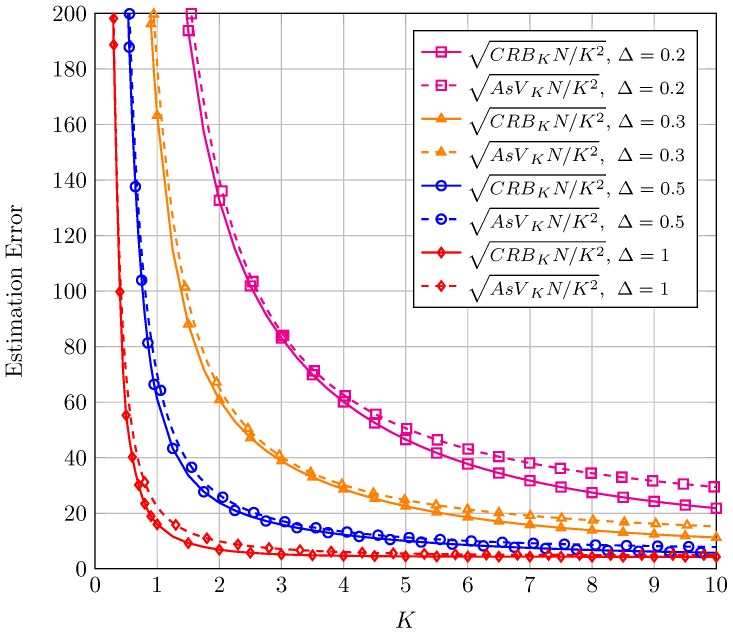
Sqrt-normalized CRB${}_{K}$ and AsV${}_{K}$ as a function of *K*, for different values of Δ.

In Figure 1, the sqrt-normalized ${\text{CRB}}_{K}$ and the sqrt-normalized ${\text{AsV}}_{K}$ are plotted as a function of *K*. Different plots correspond to different values of the unknown parameter Δ. Focusing first on the CRB, see that the sqrt-normalized ${\text{CRB}}_{K}$ grows as Δ decreases. This means that the error bound in estimating the ratio of the LOS components power to the diffuse components power is higher when there is only one LOS component. The estimation can improve as the two LOS components become of similar magnitude. See also that the error bound decreases drastically as *K* increases from 0 to a moderate value (around K=2 for Δ<0.5); this is, as the power of the diffuse components diminishes. Finally, for larger *K* the error converges to a constant value as *K* goes to infinity. The value of the sqrt-normalized ${\text{AsV}}_{K}$ is remarkably close to the sqrt-normalized ${\text{CRB}}_{K}$ for the entire considered range of parameters. This means that the proposed estimator of the *K* parameter is almost asymptotically efficient.

**Figure 2:**
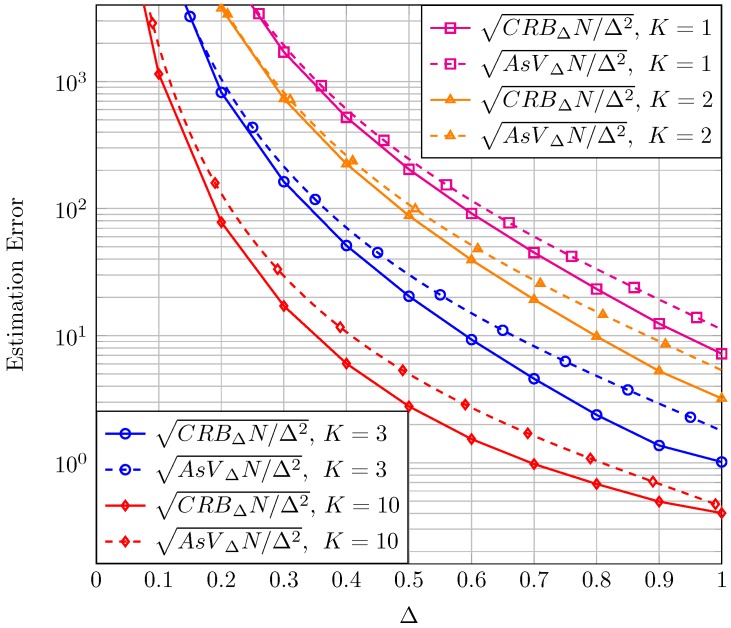
Sqrt-normalized CRB${}_{\Delta }$ and AsV${}_{\Delta }$ as a function of Δ, for different values of *K*.

Proceeding in an analogous manner, in Figure 2, we have represented the sqrt-normalized ${\text{CRB}}_{\Delta }$ and the sqrt-normalized ${\text{AsV}}_{\Delta }$ as a function of Δ. Different plots correspond to different values of the unknown parameter *K*. With regard to the sqrt-normalized ${\text{CRB}}_{\Delta }$, we notice once again that the estimation improves when *K* grows large. We also observe that for a given *K*, the sqrt-normalized ${\text{CRB}}_{\Delta }$ increases as Δ→0; this implies that it is difficult to determine the relative amplitudes of the two LOS components as one of them vanishes. The converse can be argued when both tend to be equal (i.e., Δ→1). We also see that the values of the sqrt-normalized ${\text{AsV}}_{\Delta }$ are relatively close to the sqrt-normalized ${\text{CRB}}_{\Delta }$ for the range of parameters analyzed, but not as close as when estimating *K*.

## 5. Effect of a Finite Number of Observations

**Figure 3:**
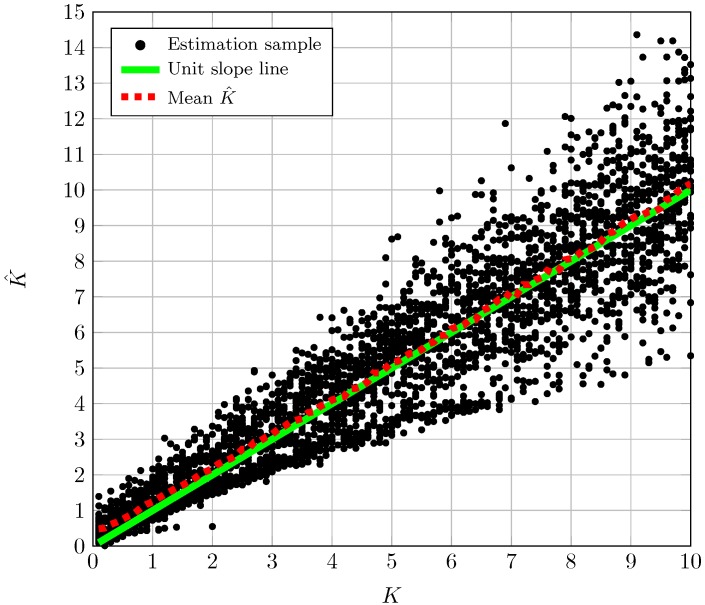
Samples of $\hat{K}$ using Equation (14) vs. true K for Δ=0.5.

**Figure 4:**
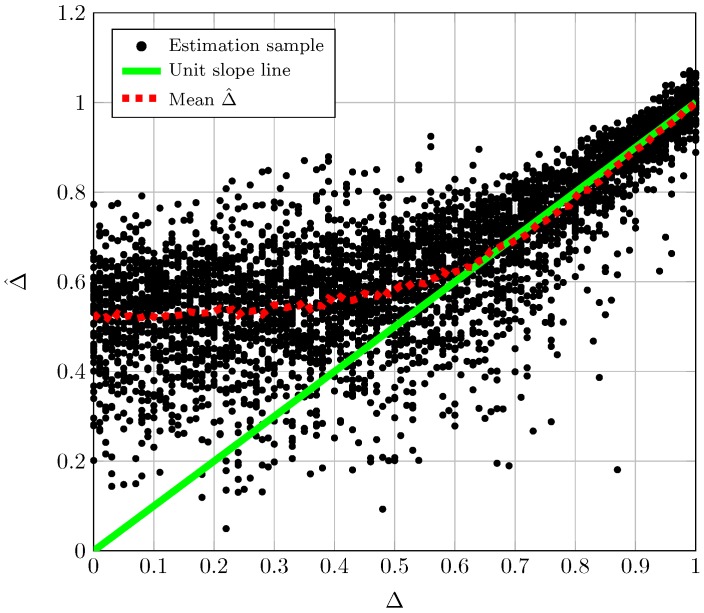
Samples of $\hat{\Delta }$ Equation (16) vs. true Δ for K=10.

The performance of the proposed estimators for finite *N* is studied by resorting to Monte-Carlo simulations. For every fixed pair of *K* and Δ, we have generated 500 sets of *N* i.i.d. realizations of the TWDP random variable. As an initial sanity check, Figure 3 shows the estimated *K* using Equation (14) vs. the true *K* value for a fixed value of Δ. The dashed line on the graphic results from averaging the estimate over the trials while the solid lined corresponds to the unit slope line that serves as a reference. From this type of plot we can extract qualitative results about the behavior of the estimators. As can be seen in Figure 3, the estimator $\hat{K}$ shows a reduced bias for the range of *K* values shown in the figure. See also that the dispersion of the estimated values grows with growing *K*. Accordingly, Figure 4 shows the estimated Δ using Equation (16) vs. the true Δ value for a fixed value of *K*. In this case the estimator shows a growing bias and dispersion of values as Δ decreases.

In order to perform a quantitative analysis of the estimators performance we have computed the sample mean squared errors ${\text{MSE}}_{K}$ and ${\text{MSE}}_{\Delta }$ where ${\text{MSE}}_{K}=\frac{1}{500}\sum_{i=1}^{500}\epsilon^{2}$ with $\epsilon =\hat{K}-K$ and where ${\text{MSE}}_{\Delta }$ is defined in an analogous way. The double normalization used with the CRB and AsV is also applied to the MSE, and hence term sqrt-normalized ${\text{MSE}}_{K}$ denotes the square root of the MSE in the estimation of K normalized to both K and N and corresponds to $\sqrt{\frac{{\text{MSE}}_{K}N}{K^{2}}}$. The same holds for sqrt-normalized ${\text{MSE}}_{\Delta }$.

**Figure 5:**
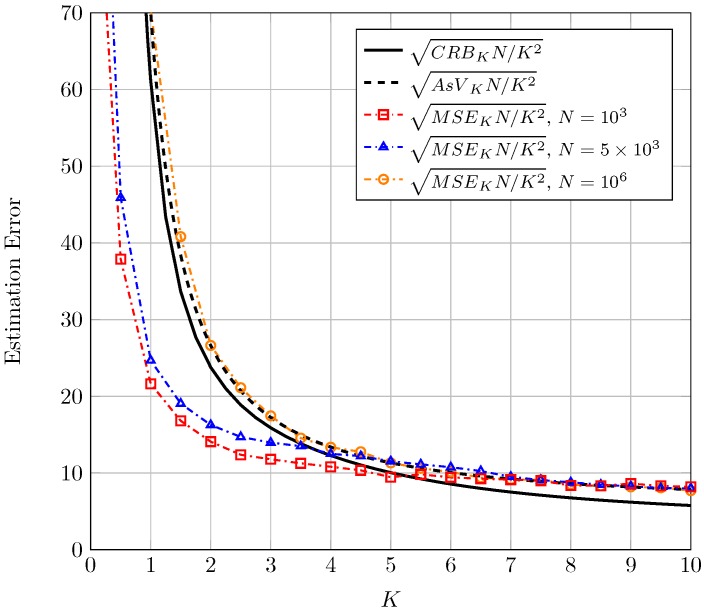
Sqrt-normalized MSE${}_{K}$ for the estimation of *K* using Equation (14) for Δ=0.5 and different number of observations *N*. Also shown are the corresponding sqrt-normalized AsV${}_{K}$ and sqrt-normalized CRB${}_{K}$.

**Figure 6:**
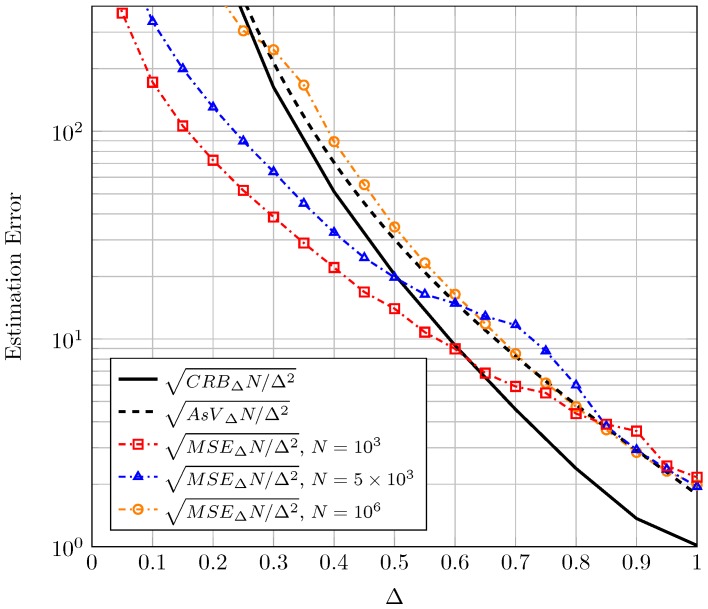
Sqrt-normalized MSE${}_{\Delta }$ for the estimation of Δ using Equation (16) for K=3 and different number of observations *N*. Also shown are the corresponding sqrt-normalized AsV${}_{\Delta }$ and sqrt-normalized CRB${}_{\Delta }$.

In Figure 5, the sqrt-normalized ${\text{MSE}}_{K}$ using Equation (14) is plotted as a function of *K* for Δ=0.5 and different values of *N*. Accordingly, in Figure 6, the sqrt-normalized ${\text{MSE}}_{\Delta }$ using Equation (16) is plotted as a function of Δ for K=3 and different values of *N*. In both figures the corresponding sqrt-normalized CRB and AsV have been included as a reference.

See that for a very high number of observations ($N=10^{6}$) the sqrt-normalized MSE is close to the sqrt-normalized AsV in both cases, as expected. As *N* decreases, the sqrt-normalized MSE falls below the sqrt-normalized CRB for values of K≲4 in Figure 5 and for values of Δ≲0.6 in Figure 6. The reason for this is that the bias of the proposed estimators grows when decreasing *N*. It is well-known that the CRB sets a limit on the variance of unbiased estimators. However, in case the estimators are biased the CRB is not practical and the MSE (which accounts for both variance and bias) may take a lower value. This behavior is also observed in the moment-based Rice parameter estimators proposed in the literature for values of the Rician parameter *K* close to zero and finite *N* (e.g., see Figure 5 in [6]). Although a CRB and a MSE bound for biased estimators can be also resorted [23], they are impractical since they require an *a priori* choice of the bias gradient. Although not explicitly included in Figure 5 and Figure 6 a similar behavior is observed for other values of *K* and Δ. Due to the double normalization applied to the sqrt-normalized MSE, care must be taken when interpreting the relative values of the different sqrt-normalized MSE curves shown in Figure 5 and Figure 6. For instance, in Figure 6, the sqrt-normalized MSE curve corresponding to $N=5\times 10^{3}$ takes a higher value than that of $N=10^{3}$ in most of the range where Δ varies. De-normalizing the sqrt-normalized MSE with respect to *N* reveals that the sqrt-normalized MSE with $N=5\times 10^{3}$ is lower than that with $N=10^{3}$ for the whole range of Δ; i.e., the estimation error is in fact lower for higher number of observations, as expected.

**Figure 7:**
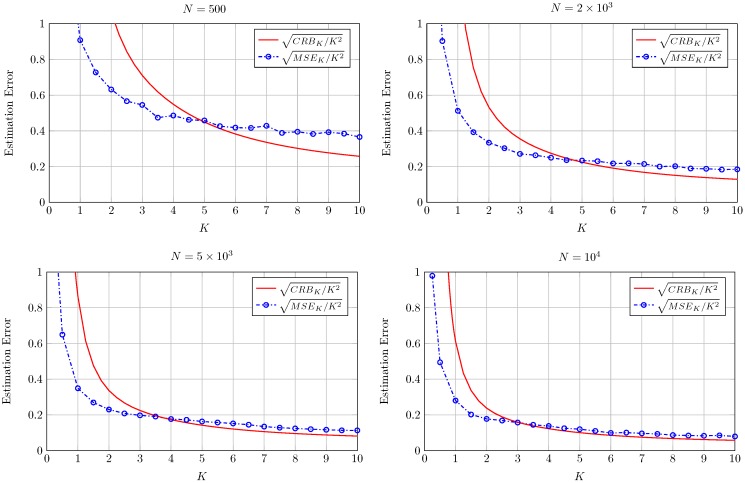
Square root of CRB${}_{K}$ and MSE${}_{K}$ normalized by K for the estimation of *K* using Equation (14) for Δ=0.5 and different number of observations *N*.

**Figure 8:**
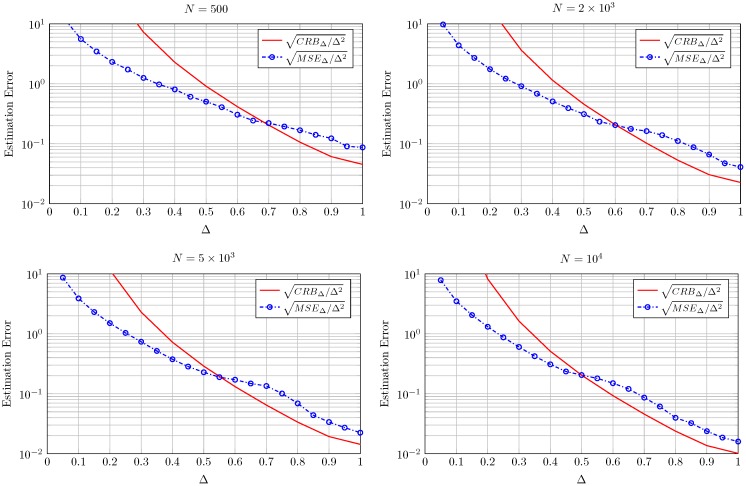
Square root of CRB${}_{\Delta }$ and MSE${}_{\Delta }$ normalized by Δ for the estimation of Δ using Equation (16) for K=3 and different number of observations *N*.

This fact is explicitly shown in Figure 7 and Figure 8 where in this case both the CRB and MSE are not normalized by *N*. See how both the CRB and the MSE decrease as *N* grows from 500 to $10^{4}$ for all the values of *K* and Δ in Figure 7 and Figure 8, respectively.

## 6. Conclusions

In this work, the joint estimation of the two parameters of the TWDP fading model has been addressed. A simple but accurate closed-form moment-based estimator for both parameters was used for the estimation. Results show that the proposed estimators operate relatively close to the CRB for a wide range of parameter values. However, the estimator of *K* is comparatively better than the estimator of Δ, as the AsV is closer to the CRB. The results here presented can set the underpinnings for alternative solutions for the parameter estimation of the TWDP fading model.

## Appendix A: Expressions for the Higher Order Moments *μ*_8_, *μ*_10_ and *μ*_12_

Computation of the covariance matrix Σ in Equation (19) requires the use of the higher order moments $\mu_{8}$, $\mu_{10}$ and $\mu_{12}$. By direct integration of Equation (6), the following expressions for the higher order moments of the signal envelope can be obtained as

(A1) $$\begin{array}{c} \begin{array}{cc} \mu_{8}=\frac{\Omega^{4}}{8{\left(1+K\right)}^{4}} & (192+K(8(4+K)(24+K(12+K))+\\ & 24K\left(2+K\right)\left(6+K\right)\Delta^{2}+3K^{3}\Delta^{4})) \end{array} \end{array}$$

(A2) $$\begin{array}{c} \begin{array}{cc} \mu_{10}=\frac{\Omega^{5}}{8{\left(1+K\right)}^{5}} & (960+K(8(20+K(10+K))(30+K(15+K))+\\ & 40K\left(60+K\left(60+K\left(15+K\right)\right)\right)\Delta^{2}+15K^{3}\left(5+K\right)\Delta^{4})) \end{array} \end{array}$$

(A3) $$\begin{array}{c} \begin{array}{cc} \mu_{12}=\frac{\Omega^{6}}{16{\left(1+K\right)}^{6}} & (16(720+K(6+K)(720+K(780+K(270+K(30+K)))))+\\ & 120K^{2}\left(360+K\left(480+K\left(180+K\left(24+K\right)\right)\right)\right)\Delta^{2}+\\ & 90K^{4}\left(30+K\left(12+K\right)\right)\Delta^{4}+5K^{6}\Delta^{6}) \end{array} \end{array}$$
